# Proinflammatory and proapoptotic markers in relation to mono and di-cations in plasma of autistic patients from Saudi Arabia

**DOI:** 10.1186/1742-2094-8-142

**Published:** 2011-10-15

**Authors:** Afaf K El-Ansary, Abir G Ben Bacha, Laila Y Al-Ayadhi

**Affiliations:** 1 Biochemistry Department, Science College, King Saud University, P.O box 22452, Zip code 11495, Riyadh, Saudi Arabia; 2 Autism Research and Treatment Center, Riyadh, Saudi Arabia; 3 Shaik AL-Amodi Autism Research Chair, King Saud University, Riyadh, Saudi Arabia; 4 Department of Physiology, Faculty of Medicine, King Saud University, Riyadh, Saudi Arabia

**Keywords:** Ions, Caspase3, IL6, TNFα, Autism

## Abstract

**Objectives:**

Autism is a developmental disorder characterized by social and emotional deficits, language impairments and stereotyped behaviors that manifest in early postnatal life. This study aims to clarify the relationship amongst absolute and relative concentrations of K^+^, Na^+^, Ca^2+^, Mg^2+ ^and/or proinflammatory and proapoptotic biomarkers.

**Materials and methods:**

Na^+^, K^+^, Ca^2+^, Mg^2+^, Na^+^/K^+^, Ca^2+^/Mg^2+ ^together with IL6, TNFα as proinflammatory cytokines and caspase3 as proapoptotic biomarker were determined in plasma of 25 Saudi autistic male patients and compared to 16 age and gender matching control samples.

**Results:**

The obtained data recorded that Saudi autistic patients have a remarkable lower plasma caspase3, IL6, TNFα, Ca^2+ ^and a significantly higher K^+ ^compared to age and gender matching controls. On the other hand both Mg^2+ ^and Na^+ ^were non-significantly altered in autistic patients. Pearson correlations revealed that plasma concentrations of the measured cytokines and caspase-3 were positively correlated with Ca^2+ ^and Ca^2+^/K^+ ^ratio. Reciever Operating Characteristics (ROC) analysis proved that the measured parameters recorded satisfactory levels of specificity and sensitivity.

**Conclusion:**

Alteration of the selected measured ions confirms that oxidative stress and defective mitochondrial energy production could be contributed in the pathogenesis of autism. Moreover, it highlights the relationship between the measured ions, IL6, TNFα and caspase3 as a set of signalling pathways that might have a role in generating this increasingly prevalent disorder. The role of ions in the possible proinflammation and proapoptic mechanisms of autistics' brains were hypothesized and explained.

## Introduction

Children with Autism Spectrum Disorders (ASD) have impairments in three core domains: socialization, communication, and restricted interests and repetitive behaviors [1-4]. Researchers have reported that psychiatric comorbidity in ASD ranges from 41% to 70% [5,6].

Although the etiology of the disorder is unknown, recent studies have suggested that the susceptibility to autism is clearly attributable to genetic factors [7,8]. In addition, emerging evidence points to inflammatory and apoptotic mechanisms being responsible for certain neuropsychiatric disorders including autism. Vargas et al. [9] suggested neuroinflammatory processes are present in the autistic brain by showing that transforming growth factor (TGF)α1, macrophage chemoattractant protein (MCP) 1, interleukin (IL)6 and IL10 are increased in the brain of autistic subjects. A number of studies have also shown that inflammatory cytokines including tumor necrosis factor (TNF)α, interferon (IFN)α, IL1α, IL6, IL8 and IL12 are elevated in blood mononuclear cells, serum, plasma and cerebrospinal fluid (CSF) of autistic subjects [9-16].

The mechanisms of apoptosis induction are complex and not fully known, but some key events are identified that appear essential for the cell to enter apoptosis. The role of specific ions in the apoptotic process is slowly being revealed. Changes in intracellular Ca^2+ ^have long been associated with apoptotic neuronal cell death. Ca^2+ ^ionophores have been shown to induce ultrastructural changes, such as cell shrinkage, chromatin condensation, and DNA fragmentation, consistent with apoptosis [17-20]. Increased Ca^2+ ^has been linked to processes occurring during apoptosis including caspase activation.

One key event in apoptosis is loss of intracellular potassium ions (K^+^). Depletion of K^+ ^is necessary for cells to shrink, activate caspases and degrade DNA [21-23], events that in turn lead to further characteristic apoptotic changes such as membrane blebbing and formation of apoptotic bodies. Apoptosis due to forced loss of intracellular K^+ ^can be induced by ionophores or K^+ ^channel activators [24-26]. In addition, Yu et al. [25,27] have also shown that the outward K^+ ^current that ensues from N-methyl-D-aspartate receptor activation has also been shown to induce apoptotic changes in cultured hippocampal neurons.

Just as with increased Ca^2+^and K^+ ^efflux, the importance of sodium (Na^+^) entry in inducing neuronal injury and death in response to pathophysiologic conditions, such as hypoxia, has been well established [28-34]. Moreover, Banasiak et al. [35] proved that blocking Na^+ ^entry in hypoxia-exposed neurons reduced the proportion of DNA fragmentation and reduced apoptotic cell.

Magnesium (Mg^2+^) has a profound effect on neural excitability; the most characteristic signs and symptoms of Mg^2+ ^deficiency are produced by neural and neuromuscular hyperexcitability [36]. Iotti and Malucelli [37] clarify the functional relationship between energy metabolism and free [Mg^2+^], providing evidence that brain cells cytosolic [Mg^2+^] is regulated to equilibrate any changes in rapidly available free energy. Moreover, it has also been shown that the measurement of brain Mg^2+ ^can help in the differential diagnosis of neurodegenerative diseases sharing common clinical features.

The immune system has been postulated to play an important role in the etiology of autism. Investigators have proposed infectious, autoimmune, and cytokine-related etiologies.

These information initiate our interest to measure concentrations of Na^+^, K^+^, Ca^2+^, Mg^2+ ^together with caspase3 as a proapoptotic marker, IL6 and TNFα as proinflammation markers in the plasma of autistic patients from Saudi Arabia in an attempt to understand the role and relationship of these biochemical parameters in the etiology of autism and its commonly related psychiatric conditions.

## Material and methods

### Subjects and methods

The study protocol followed the ethical guidelines of the most recent Declaration of Helsinki (Edinburgh, 2000). All subjects enrolled in the study (25 autistic male patients and 16 age and gender matched controls) had written informed consent provided by their parents and assented to participate if developmentally able. They were enrolled through the ART Center (Autism Research & Treatment Center) clinic (Riyadh, Saudi Arabia). The ART Center clinic sample population consisted of children diagnosed on the ASD. The diagnosis of ASD was confirmed in all subjects using the Autism Diagnostic Interview-Revised (ADI-R) and the Autism Diagnostic Observation Schedule (ADOS) and 3DI (Developmental, dimensional diagnostic interview). The ages of all autistic children who participated were between the ages of 4 and 12 years old. All were simplex cases. All are negative for fragile × gene study. The control group recruited from Well baby Clinic at King Khaled University hospital with mean age 4-11 year old. Subjects were excluded from the investigation if they had organic aciduria, dysmorphic features, or diagnosis of Fragile × or other serious neurological (e.g., seizures), psychiatric (e.g., bipolar disorder) or known medical conditions. All participants were screened via parental interview for current and past physical illness. Children with known endocrine, cardiovascular, pulmonary, liver, kidney or other medical disease were excluded from the study. None of the recruited autistic patients were on special diets or alternative treatments.

### Ethics approval and consent

A written consent was obtained from the parents of each individual case, according to the guidelines of the ethical committee of King Khalid Hospital, King Saud University.

### Blood samples

After overnight fast, 10 ml blood samples were collected from both groups in test tubes containing sodium heparin as anticoagulant. Tubes were centrifuged at 3500 rpm at room temperature for 15 minutes, plasma was obtained and deep freezed (at -80°C) until analysis time.

### Measurement of calcium

The UDI (United Diagnostics Industry, Saudi Arabia) Ca^2+ ^procedure is based on the reaction of Ocresolphthalein complexone (O-CPC) with Ca^2+ ^to form a chromogenic complex that absorbs light which is measured photometrically at 575 nm. Mg^2+ ^interference is prevented by sequestration with 8-hydroxyquinoline. 2-Ethylaminoethanol is used to establish the reaction pH at 12. Dimethyl sulfoxide is used to lower the dielectric constant of the reaction mixture and to repress the ionization of cresolphthalein complexone [38].

### Measurement of potassium

K^+ ^reacts with sodium tetra phenyl boron in a protein free alkaline medium to produce a colloidal suspension [39]. The turbidity which is proportional to the K^+ ^concentration in the range of 2-7 mmol/L was measured against blank. The concentration was calculated using a typically treated standard solution of K^+ ^chloride in Bovine albumin equivalent to 4 mol/L.

### Measurement of sodium

Plasma Na^+ ^was measured according to the method of Tietz [40] using a diagnostic kit, a product of UDI in which Na^+ ^was determined via Na+ dependent β-galactosidase activity using O-nitrophenyl-β, D-galactopyranoside.

### Measurement of magnesium

The UDI method stems from the original work of Lindstrom and Diehl [41] using calmagite, 1-(1-hydroxy-4-methyl-2- phenylazo)-2-naphthol-4-sulfonic acid, as the complexometric reagent. Ca^2+ ^is masked by sequestration with strontium ethylene-bis-(oxyethylenenitrilo)-tetra acetate (EGTA Sr) [42]. A surfactant system has been utilized to overcome protein interference. Mg^2+ ^form a colored complex with calmagite in alkaline medium to produce a red complex that absorbs light which is measured spectrophotometrically at 530 nm. The absorbance of the red complex is directly proportional to the concentration of Mg^2+ ^in the sample.

### Statistical analysis

A SPSS (Statistical Package for the Social Sciences) computer program was used. Results were expressed as mean ± S.D. and all statistical comparisons were made by means of independent t-test with P ≤ 0.05 was considered significant. ROC analysis was performed. Area under the curve, cutoff values together with degree of specificity and sensitivity were calculated.

## Results

**Figure 1:**
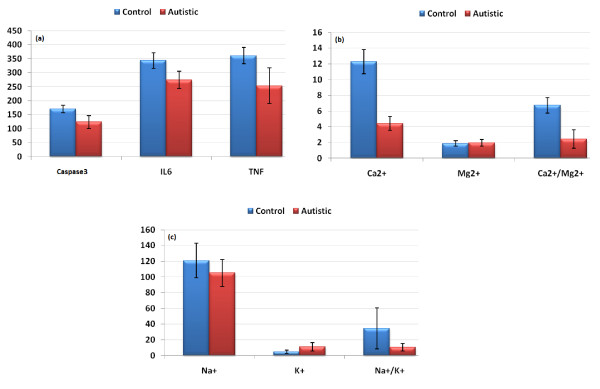
**Mean with the standard error bars of measured Caspase3, IL6 and TNFα **(a)**, Ca^2+^, Mg^2+ ^and Ca^2+^/Mg^2+^**(b)**, and Na^+^, K^+ ^and Na^+^/K^+^**(c) **in autistic patients (N = 25) compared to age and gender matching controls (N = 16)**. Caspase3 concentration is expressed as ng/mL plasma and IL6 and TNFα concentrations are expressed as pg/mL plasma. Na^+^, K^+^, Mg^2+ ^and Ca^2+ ^concentrations are expressed in mmol/L plasma.

**Figure 2:**
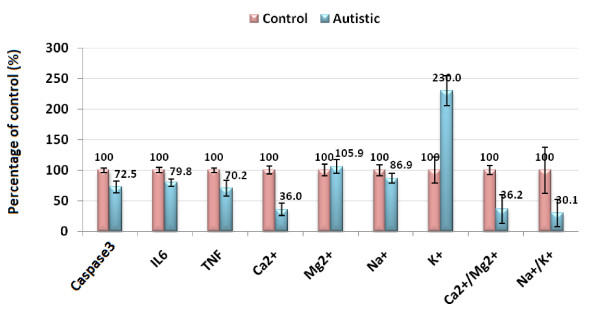
**Percentage change in caspase3, IL6, TNFα, Ca^2+^, Mg^2+^, Na^+^, K^+^, Ca^2+^/Mg^2+ ^and Na^+^/K^+ ^of autistic patients (N = 25) compared to age and gender matching controls (N = 16)**.

**Figure 3:**
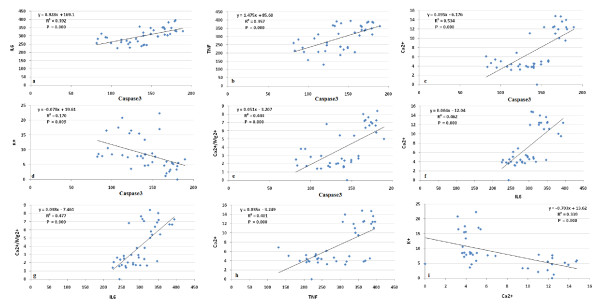
**Pearson correlations between the measured parameters with best fit line curve**: **(a) **Caspase3 and IL6 (positive correlation); **(b)**: Caspase3 and TNFα (positive correlation); **(c)**: Caspase3 and Ca^2+ ^(positive correlation); **(d)**: Caspase3 and K^+ ^(negative correlation); **(e)**: Caspase3 and Ca^2+^/Mg^2+ ^(positive correlation); **(f)**: IL6 and Ca^2+ ^(positive correlation); **(g)**: IL6 and Ca^2+^/Mg^2+ ^(positive correlation), **(h)**: TNFα and Ca^2+ ^(positive correlation); (**i)**: Ca^2+ ^and K^+ ^(negative correlation).

**Figure 4:**
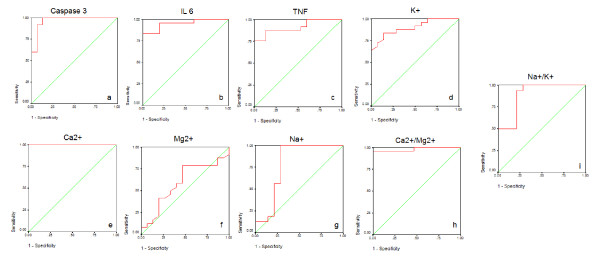
**ROC curves showing area under the curves, specificity and sensitivity of caspase3 **(a)**, IL6 **(b)**, TNFα **(c)**, K^+^**(d) **Ca^2+^**(e)**, Mg^2+^**(f)**, Na^+^**(g) **Ca^2+^/Mg^2+^**(h) **and Na^+^/K^+^**(i) **in autistic patients (N = 25)**.

Table 1 and Figure 1 demonstrate concentrations of the measured parameters in plasma of autistic patients compared to control. Concentrations of caspase3, IL6 and TNFα were significantly lower in children with autism compared to control. In contrast, K^+ ^was significantly raised in plasma samples from children with autism compared to age and gender matching controls recording 2.3 fold higher values. In addition, Ca^2+^, Ca^2+^/Mg^2+ ^and Na^+^/K^+ ^ratio were significantly lower in autistic compared to control with the latter showing almost 3 fold lower values. Figure 2 shows the percentage changes of the measured parameters in autistics relative to control subjects. It could be easily seen that caspase3, IL6 and TNFα recorded more or less the average % decrease with values of -27.5,-20.2 and -29.8. Among the measured elements K^+ ^recorded the most remarkable percentage increase recording value of 130% higher concentration in autistic compared to control with concomitant decrease in Na^+^/K^+ ^ratio of 69.9% decrease. Ca^2+^/Mg^2+ ^ratio recorded 63.8% lower values in control. Absolute values of Na^+ ^and Mg^2+ ^recorded the lowest percentage changes recording 13.1% and 5.9% increase, respectively. Table 2 and Figure 3 show the significantly positive and negative correlated parameters. Out of the 27 correlations recorded in table 3, the most significantly correlated parameters were selected to be presented in Figure 3. Table 3 together with Figure 4 show ROC analysis of the measured parameters. It could be easily noticed that most of the measured parameters recorded satisfactory values of sensitivity and specificity with the exception of Mg^2+ ^and Na^+ ^which show low specificity values.

**Table 1:** Caspase3, IL6, TNFα, Ca^2+^, Mg^2+^, Na^+ ^and K^+ ^concentrations and Ca^2+^/Mg^2+ ^and Na^+^/K^+ ^ratios in plasma of autistic patients (N = 25) compared to age and gender matching controls (N = 16)

Parameters	Groups	**Min**.	**Max**.	**Mean ± S.D**.	P value
**Caspase3 (ng/ml)**	Control	135.54	189.47	170.17 ± 13.05	> 0.001
		
	Autistic	81.94	158.28	123.40 ± 23.37	
	
**IL6** **(pg/ml)**	Control	303.18	394.41	343.34 ± 28.16	
		
	Autistic	225.42	347.41	273.95 ± 30.82	
	
**TNFα** **(pg/ml)**	Control	306.53	395.66	360.85 ± 29.05	
		
	Autistic	129.44	381.28	253.16 ± 64.07	
	
**Ca^2+^** **(mmol/L)**	Control	9.49	14.77	12.29 ± 1.53	
		
	Autistic	3.17	6.85	4.42 ± 0.87	

**Mg^2+^** **(mmol/L)**	Control	1.42	2.47	1.86 ± 0.35	0.411
		
	Autistic	1.00	2.76	1.97 ± 0.43	

**Na^+^** **(mmol/L)**	Control	76.20	139.92	120.92 ± 21.94	0.036
		
	Autistic	65.18	123.69	105.06 ± 17.43	

**K^+^** **(mmol/L)**	Control	1.20	7.90	4.76 ± 2.04	> 0.001
		
	Autistic	3.60	22.30	10.95 ± 5.26	
	
**Ca^2+^/Mg^2+^**	Control	5.01	8.41	6.74 ± 0.99	
		
	Autistic	1.40	6.82	2.44 ± 1.15	

**Na^+^/K^+^**	Control	10.45	109.14	34.55 ± 26.01	0.004
		
	Autistic	4.15	19.57	10.41 ± 4.73	

**Table 2:** Pearson correlation test between the measured parameters

Parameters	R (Person Correlation)		**Sig**.
**Caspase3 ~ IL6**	0.627	+	> 0.01
	
**Caspase3 ~ TNFα**	0.598	+	
	
**Caspase3 ~ Ca^2+^**	0.731	+	
	
**Caspase3 ~ Na^+^**	0.486	+	
	
**Caspase3 ~ K+**	-0.412	-	
	
**Caspase3 ~ Ca^2+^/Mg^2+^**	0.666	+	
	
**Caspase3 ~ Na^+^/K^+^**	0.459	+	
	
**IL6 ~ TNFα**	0.469	+	
	
**IL6 ~ Ca^2+^**	0.680	+	
	
**IL6 ~ Na^+^**	0.505	+	
	
**IL6 ~ K^+^**	-0.423	-	
	
**IL6 ~ Ca^2+^/Mg^2+^**	0.691	+	
	
**IL6 ~ Na^+^/K^+^**	0.551	+	
	
**TNFα ~ Ca^2+^**	0.633	+	
	
**TNFα ~ Ca^2+^/Mg^2+^**	0.521	+	
	
**Ca^2+ ^~ K^+^**	-0.582	-	
	
**Ca^2+ ^~ Ca^2+^/Mg^2^+**	0.912	+	
	
**Ca^2+ ^~ Na^+^/K^+^**	0.503	+	
	
**Mg^2+ ^~ Na^+^**	-0.537	-	
	
**Mg^2+ ^~ Ca^2+^/Mg^2+^**	-0.476	-	
	
**Na^+ ^~ Ca^2+^/Mg^2+^**	0.552	+	
	
**Na^+ ^~ Na^+^/K^+^**	0.526	+	
	
**Ca^2+^/Mg^2+ ^~ Na^+^/K^+^**	0.592	+	
	
**K^+ ^~ Ca^2+^/Mg^2+^**	-0.604	-	
	
**K^+ ^~ Na^+^/K^+^**	-0.650	-	

**Na^+ ^~ K^+^**	-0.363	-	0.049

Correlation is significant at the 0.01 level (2-tailed).

**^+^**Positive Correlation

**^-^**Negative Correlation

**Table 3:** ROC analysis of Ca^2+^/Mg^2+ ^and Na^+^/K^+ ^ratios and Caspase3, IL6, TNFα, Ca^2+^, Mg^2+^, Na^+^and K^+ ^in autistic groups (N = 25)

Parameter	Area under the curve	Best Cutoff value	Sensitivity %	Specificity %
**Caspase3**	0.968	161.17	100.0%	86.7%

**IL6**	0.952	301.95	84.0%	100.0%

**TNFα**	0.915	297.67	76.0%	100.0%

**Ca^2+^**	1.000	8.17	100.0%	100.0%

**Mg^2+^**	0.592	1.76	97.2%	53.3%

**Na^+^**	0.786	124.50	100.0%	71.4%

**K^+^**	0.900	7.00	84.0%	85.7%

**Ca^2+^/Mg^2+^**	0.981	4.41	95.8%	100.0%

**Na^+^/K^+^**	0.888	17.14	93.8%	78.6%

## Discussion

Protection of the brain from injury during the fetal, neonatal and postnatal periods is of major importance owing to the significant number of infants who now survive early brain injury but develop neurodevelopmental and motor disabilities.

Table 1 and Figures 1 and 2 show the unexpected lower concentrations of caspase3, TNFα and IL6. This could be interpreted on the basis that the etiology of the fetal brain damage inflammation will involve many factors and is likely to include an increase in circulating cytokine concentrations. Rees et al. [43] have shown, for example that TNFα [44] and IL6 concentrations [45] increase within the early 6 hours of lipopolysaccharide (LPS) exposure. It has been proposed that circulating cytokines might act on cerebralendothelial cells or periventricular cells to upregulate prostaglandin synthesis, resulting in increased permeability of the blood-brain barrier [46]; thus the administration of LPS to fetal sheep results in the extravasation of plasma proteins and macrophages into the brain [46].

TNFα and IL6 are cytokines involved in cell-mediated immune response and their production has been shown to be associated with tissue inflammation and necrosis [47]. Based on these information, the recorded lower plasma concentrations of these two cytokines does not oppose with the neuroinflammatory model recently proved for autism [48]. This could help us to suggest that localized inflammation of the central nervous system may contribute to the pathogenesis of autism and that elevation of plasma cytokines could be an early event followed by infiltration of macrophages, cytokines and proapotic factors across the BBB to the brain. The lower recorded concentration of caspase3 in autistics compared to control subjects could be easily related to the decrease in TNFα. This could be supported through considering the previous report of Mundle et al. [49] which demonstrated a link between TNFα and the major effectors of its apoptotic signal, i.e. Caspase1 and 3. They identify the downstream effectors of TNFα apoptotic signalling and show a positive correlation of TNFα with Caspase3.

A major endogenous antioxidant in mammalian cells is the enzyme superoxide dismutase (SOD), which catalyzes the dismutation of the superoxide anion (O_2_^-^) into hydrogen peroxide (H_2_O_2_) and molecular oxygen (O_2_). Dimayuga et al. [50] show that overexpression of SOD1 in microglial cells leads to significant decreases in superoxide concentrations, with corresponding increases in H_2_O_2 _concentrations. They proved that the release of the proinflammatory cytokines TNFα and IL6 is significantly attenuated by overexpression of SOD1. With special consideration of the effect of population, the recorded lower concentrations of TNFα and IL6 in autistic patients as subjects of the present study compared to controls could be related to the overexpression of SOD previously reported as metabolic biomarker in Saudi autistic patients [51].

Table 1 and Figure 2 demonstrate that autistic patients from Saudi Arabia recorded lower concentrations of plasma Ca^2+^. This could find a support through considering the work of Shearer et al. [52] in which they observed lower Ca^2+ ^concentrations in the hair of autistic population and that of Krey and Dolmetsch [53] in which they proved that some forms of autism are caused by failures in activity-dependent regulation of neural development due to mutations of several voltage-gated and ligand-gated ion channels that regulate neuronal excitability and Ca^2+ ^signalling. On the other hand, the recorded lower concentration of Ca^2+ ^is not in accordance with the recent work of Laura et al. (2011) [54] which reported higher Ca^2+ ^concentrations in plasma of Italian autistic patients compared to age and gender matching controls. The reduced plasma Ca^2+ ^concentrations of the present study could be associated with high intracellular brain Ca^2+ ^in autistics compared to control subjects. This suggestion could be supported with the recent evidence from post-mortem studies of autistic brains which points toward abnormalities in mitochondrial function as possible downstream consequences of dysreactive immunity and altered Ca^2+ ^signalling [55]. Low plasma Ca^2+ ^and the speculated high brain Ca^2+ ^concentration could be easily correlated to the oxidative stress previously recorded in Saudi autistic patients [56] as elevated brain Ca^2+ ^is recently related to ROS generation. Mitochondrial aspartate/glutamate carrier (AGC1), isoform predominantly expressed in the brain, heart and skeletal muscle, is known to play a pivotal role in energy metabolism and is regulated by neurone intracellular Ca^2+^[57,58]. This carrier was found to be approximately three-fold higher in brain homogenates from each of six autistic patients compared to their matched controls. This could support the lower plasma Ca^2+ ^concentrations recorded in the present study. Moreover, direct fluorimetric measurements of Ca^2+ ^concentrations in the post-mortem mitochondrial supernatant confirmed significantly higher Ca^2+ ^concentrations in brain of autistics [55].

This suggested increased influx of blood-to-brain Ca^2+ ^could be easily related to the loss of amyloid beta (Aβ) equilibrium between the brain and blood which may lead to failure of drawing out Aβ from the brain across the blood brain barrier (BBB) as a mechanism for Aβ accumulation in Saudi autistics [Al-Ayahdi L, Ben Bacha A, Kotb M, El-Ansary A: **A novel study on amyloid β peptide 40, 42 and 40/42 ratio in Saudi autistics**, Submitted]. Vitamin E which is known to attenuate A β-induced apoptosis despite Ca^2+ ^accumulation in brain cells is significantly lower in Saudi autistic patients [51]. This could support the suggested mechanism relating Aβ and Ca^2+^- induced apoptosis in brain cells of Saudi autistics.

Table 1 and Figure 1 demonstrate K^+ ^concentrations in plasma of autistic and control subjects. It could be easily noticed that autistic patients recorded raised concentrations of K^+ ^compared to controls. This could be attributed to the altered Na^+^/K^+ ^ATPase activity previously reported by El-Ansary et al. [56], which may represent an important neurotoxic mechanism for neurons.

The recorded higher plasma concentrations of K^+ ^which reflect the remarkable higher rate of k^+ ^efflux from brain to blood in autistic patients could be easily related to the significant lower Ca^2+^, the unchanged Na^+^, lower Ca^2+^/Na^+ ^ratios and to the speculated higher brain caspase3 activity. Xiao et al. [59] showed previously that activation of the N-methyl-D-aspartic acid (NMDA) subtype of glutamate receptors in a low Ca^2 ^and Na^+ ^condition induced apoptotic neuronal death, and that the K^+ ^efflux via NMDA receptor channels was likely a key event in NMDA-induced apoptosis. This postulation could be supported by Pigozzi et al. [60] who proved that entry of Ca^2+ ^into neuron cells can accelerate apoptosis by accelerating the expression of growth arrest and DNA Damage inducible gene 153 (GADD153) and by inducing a prolonged efflux of K^+ ^out of the cell. This is in good agreement with the elevated K^+ ^and the reduced Ca^2+ ^concentrations in plasma of autistic patients compared to controls as a report of the present study. Moreover, the significantly impaired Ca^2+ ^and K^+ ^concentrations in plasma of autistic patients could be easily related to the postulated increase of brain cytokines (TNFα and IL6) after infiltration from plasma to brain. Experimental evidence demonstrates that ion channels are targeted by cytokines, which can specifically modulate their function [61] and TNFα was associated with the remarkable Ca^2+ ^influx from blood to brain [62]. These suggested mechanisms of the alteration of the studied parameters could be supported through the obtained Pearson correlations presented in table 3 and Figure 3.

ROC analysis presented in Figure 4, support the previous discussion and suggestions which based on the obtained data. Most of the measured parameters recorded AUC near 1 and satisfactory levels of specificity and sensitivity and hence they could be used as biochemical markers for the early diagnosis of autism in Saudi population.

## Competing interests

The authors declare that they have no competing interests.

## Authors' contributions

AE designed the study and drafted the manuscript. ABB helped to draft the manuscript and performed the statistical analysis. LA provided samples and participated in the design of the study. All authors have read and approved the final manuscript.
